# Community-acquired polymicrobial pneumonia in the intensive care unit: aetiology and prognosis

**DOI:** 10.1186/cc10444

**Published:** 2011-09-14

**Authors:** Catia Cillóniz, Santiago Ewig, Miquel Ferrer, Eva Polverino, Albert Gabarrús, Jorge Puig de la Bellacasa, Josep Mensa, Antoni Torres

**Affiliations:** 1 Department of Pneumology, Institut Clinic del Tórax, Hospital Clinic of Barcelona-Institut d'Investigacions Biomèdiques August Pi i Sunyer, University of Barcelona-SGR 911-Ciber de Enfermedades Respiratorias, Villarroel 170, Barcelona 08036, Spain; 2 Thoraxzentrum Ruhrgebiet, Kliniken für Pneumologie und Infektiologie, EVK Herne und Augusta-Kranken-Anstalt Bochum, Bergstraße 26, 44791 Bochum, Germany; 3 Department of Microbiology, Hospital Clinic of Barcelona, Villarroel 170, Barcelona 08036, Spain; 4 Department of Infectious Disease, Hospital Clinic of Barcelona, Villarroel 170, Barcelona 08036, Spain

## Abstract

**Introduction:**

The frequency and clinical significance of polymicrobial aetiology in community-acquired pneumonia (CAP) patients admitted to the ICU have been poorly studied. The aim of the present study was to describe the prevalence, clinical characteristics and outcomes of severe CAP of polymicrobial aetiology in patients admitted to the ICU.

**Methods:**

The prospective observational study included 362 consecutive adult patients with CAP admitted to the ICU within 24 hours of presentation; 196 (54%) patients had an established aetiology.

**Results:**

Polymicrobial infection was present in 39 (11%) cases (20% of those with defined aetiology): 33 cases with two pathogens, and six cases with three pathogens. The most frequently identified pathogens in polymicrobial infections were *Streptococcus pneumoniae *(*n *= 28, 72%), respiratory viruses (*n *= 15, 39%) and *Pseudomonas aeruginosa *(*n *= 8, 21%). Chronic respiratory disease and acute respiratory distress syndrome criteria were independent predictors of polymicrobial aetiology. Inappropriate initial antimicrobial treatment was more frequent in the polymicrobial aetiology group compared with the monomicrobial aetiology group (39% vs. 10%, *P *< 0.001), and was an independent predictor of hospital mortality (adjusted odds ratio = 10.79, 95% confidence interval = 3.97 to 29.30; *P *< 0.001). The trend for higher hospital mortality of the polymicrobial aetiology group compared with the monomicrobial aetiology group (*n *= 8, 21% versus *n *= 17, 11%), however, was not significantly different (*P *= 0.10).

**Conclusions:**

Polymicrobial pneumonia occurs frequently in patients admitted to the ICU. This is a risk factor for inappropriate initial antimicrobial treatment, which in turn independently predicts hospital mortality.

## Introduction

Community-acquired pneumonia (CAP) remains a common and potentially life-threatening condition. Among patients hospitalised by CAP, the rates of severe CAP range from 6.6 to 16.7% [1].

The pathogens causing CAP may vary according to geographic area and underlying risk factors. Appropriate initial antimicrobial treatment has been repeatedly shown to be crucial for the outcome in severe infections. The knowledge of pathogen patterns causing CAP as the basis for the selection of such treatment is therefore crucial. Some studies have revealed that more than one causative microorganism was present in a considerable amount of cases. One of main problems for the studies on microbial aetiology in CAP is that not all microbiological tests are applied systematically for all patients. This limitation could possibly imply that the real frequency of polymicrobial aetiologies in main series is often underestimated.

The reported rates for polymicrobial aetiology, however, differ considerably between 5.7 and 38.4% [2-7]. The clinical significance of polymicrobial aetiology in CAP patients admitted to the ICU has not been specifically addressed. We therefore studied the prevalence, clinical characteristics and outcomes of severe CAP of polymicrobial aetiology in ICU patients.

## Materials and methods

### Ethics statement

The study was approved by the Ethics Committee of our institution, but written informed consent was waived due to the non-interventional design.

### Study population

The present cohort included 362 consecutive adult patients with CAP admitted to the ICU within 24 hours of admission to the emergency department in an 850-bed tertiary care university hospital (Hospital Clinic of Barcelona, Spain) between January 2003 and December 2010. The decision for admission to an ICU was made by the attending physician in all cases.

Pneumonia was defined as a new pulmonary infiltrate found on the hospital admission chest radiograph with symptoms and signs of lower respiratory tract infection. We excluded patients with immunosuppression (for example, patients with neutropaenia after chemotherapy or bone marrow transplantation, patients with drug-induced immunosuppression as a result of solid-organ transplantation or corticosteroid (> 10 mg/day) or cytotoxic therapy, and all HIV-infected patients) and healthcare-associated pneumonia patients.

### Data collection and evaluation

The following parameters were recorded at admission: age, sex, tobacco use, alcohol and drug consumption, co-morbidities (chronic respiratory disease, including chronic obstructive pulmonary disease, asthma and bronchiectasis among others, diabetes mellitus, chronic cardiovascular disease, neurological disease, chronic renal disease and chronic liver disease), antibiotic treatment in the previous 30 days before hospital admission, treatment with corticosteroids, clinical symptoms and features (fever, cough, pleuritic chest pain, dyspnoea, mental confusion and aspiration), clinical signs (blood pressure, body temperature, respiratory rate and heart rate), arterial blood gas measurements, chest radiograph findings (number of lobes affected, pleural effusion and atelectasis), laboratory parameters (haemoglobin level, white blood cell count, platelet count, serum creatinine level, C-reactive protein level and other biochemical parameters), diagnostic procedures, empiric antibiotic therapy, ventilatory support, pulmonary complications (empyema, acute respiratory distress syndrome (ARDS) criteria, pleural effusion and surgical pleural draining) and other clinical events (cardiac arrhythmias, septic shock, and acute renal failure). The duration of treatment, length of hospital stay and 30-day in-hospital mortality were noted. We also calculated the pneumonia severity index (PSI) at admission [8].

### Microbiological evaluation and diagnostic criteria

Microbiological examination was performed in sputum, urine, two samples of blood and nasopharyngeal swabs. Pleural puncture, tracheobronchial aspirates and bronchoalveolar lavage (BAL) fluid, when available, were collected. Conventional tests were used to evaluate the presence of bacterial, parasitic and fungal agents, and of respiratory viruses. Sputum, Bronchial aspirate sample (BAS) and BAL specimens were stained using the Gram and Ziehl-Neelsen methods for bacterial and mycobacteria detection, respectively. In BAL samples, the following additional stains were used: May-Grünwald Giemsa for fungal detection and cellular differential count, and Gomori methenamine silver for *Pneumocystis jirovecii*. Sputum and pleural fluid samples were qualitatively cultured for bacterial pathogens, fungi and mycobacteria. Bronchial aspirate sample (BAS) and BAL samples were homogenised and processed for quantitative culture by serial dilutions for bacterial pathogens; undiluted cultures for *Legionella *spp., fungi and mycobacteria were also carried out.

Nasopharyngeal swabs and BAL specimens were processed for antigen detection by immunofluorescence assay and for isolation of viruses in cell culture (influenza virus A, influenza virus B, human parainfluenza viruses 1 to 3, adenovirus and respiratory syncytial virus). In addition, two independent multiplex-nested RT-PCR assays able to detect from 1 to 10 copies of viral genomes were performed for the diagnostics of respiratory viruses. One RT-PCR assay detected influenza virus types A, B and C, respiratory syncytial viruses A and B, and adenovirus. Another RT-PCR assay studied parainfluenza viruses 1, 2, 3 and 4, coronaviruses 229E and OC43, rhinoviruses and enteroviruses. All positive results were subsequently confirmed by a second independent assay.

Sputum and blood samples were obtained for bacterial culture before the start of antibiotic therapy in the emergency department. Nasopharyngeal swab for respiratory virus detection and urine samples for *Streptococcus pneumoniae *and *Legionella pneumophila *antigen detection were obtained within 24 hours after hospital admission. Blood samples for serology of atypical pathogens and respiratory virus were taken at admission and within the third and sixth week thereafter.

### Criteria for aetiological diagnosis

The aetiology was considered definite if one of the following criteria was met: blood culture positive (in the absence of an apparent extrapulmonary focus); positive bacterial culture of pleural fluid or transthoracic needle aspiration samples; elevated serum levels of IgM against *Chlamydophila pneumoniae *(≥ 1:64), *Coxiella burnetii *(≥ 1:80) and *Mycoplasma pneumoniae *(any positive titre); seroconversion (that is, a fourfold increase in IgG titres) for *C. pneumoniae *and *L. pneumophila *> 1:128, *C. burnetii *> 1:80 and respiratory viruses (influenza viruses A and B, parainfluenza viruses 1 to 3, respiratory syncytial virus and adenovirus); positive urinary antigen for *L. pneumophila *(Binax Now *L. pneumophila *urinary Antigen Test; Trinity Biotech, Bray, Ireland); positive urinary antigen for *S. pneumoniae *(Binax Now *S. pneumoniae *urinary Antigen Test; Emergo Europe, The Hague, The Netherlands); bacterial growth in cultures of tracheobronchial aspirates (≥ 10^5 ^cfu/ml), in a protected specimen brush (≥ 10^3 ^cfu/ml) and in BAL (≥ 10^4 ^cfu/ml); and detection of antigens by immunofluorescence assay plus virus isolation or detection by RT-PCR testing for respiratory virus (influenza viruses A and B, parainfluenza viruses 1 to 3, respiratory syncytial virus, rhinovirus and adenovirus).

The aetiology of pneumonia was classified as presumptive when a predominant microorganism was isolated from a purulent sample (leukocytes > 25 per high-power microscopic field and few epithelial cells < 10 per high-power microscopic field) and the findings of Gram staining were compatible. For the purpose of the present study, presumptive and definitive diagnostics were analysed together.

### Definitions

Polymicrobial pneumonia was defined as pneumonia due to more than one pathogen. Severe CAP was defined when at least one major criterion or three minor criteria of the Infectious Disease Society of America/American Thoracic Society (IDSA/ATS) guidelines were present [9]. Appropriateness of empiric antibiotic treatment was defined when the isolated pathogens were susceptible *in vitro *to one of the antimicrobials administrated according to current European guidelines for microbiological susceptibility testing [10]. For *Pseudomonas aeruginosa *infection, adequate treatment needed a combination of two active antibiotics against the isolated strain.

### Statistical analysis

Categorical variables are described as frequencies and percentages, while continuous variables are presented as means and standard deviations, or as the median and interquartile range for data not normally distributed (Kolmogorov-Smirnov test). Categorical variables were compared with the chi-square test or Fisher's exact test where appropriate. Continuous variables were compared using the Student *t *test once normality was demonstrated; otherwise, the nonparametric Mann-Whitney U test was performed.

Univariate and multivariate logistic regression analyses were performed to identify variables predictive of patients with polymicrobial pneumonia (dependent variable). The independent variables analysed were: age, gender, smoking, alcohol consumption, previous antibiotic, influenza vaccine, pneumococcal vaccine, inhaled corticosteroids, systemic corticosteroids, chronic cardiovascular disease, chronic renal failure, diabetes mellitus, chronic liver disease, neurological disease, chronic pulmonary disease, fever, C-reactive protein level, white blood cell count, creatinine, PSI, multilobar infiltration, ARDS criteria, shock and mechanical ventilation. Univariate and multivariate logistic regression analyses were performed to predict 30-day mortality (dependent variable). The independent variables were the previous plus the number of aetiologies and adequacy of empirical treatment, with the exception of ARDS, shock and mechanical ventilation. Variables that showed a significant result univariately (*P *< 0.1) were included in the multivariate logistic regression backward stepwise model. The Hosmer-Lemeshow goodness-of-fit test was performed to assess the overall fit of the model [11]. All tests were two-tailed and significance was set at 5%. All analyses were performed with SPSS version 16.0 for Windows (SPSS Inc., Chicago, IL, USA).

## Results

### Patients' characteristics

During the study period, 2,200 patients were hospitalised with a diagnosis of CAP. Of these, 362 (16%) patients were admitted to the ICU. The main characteristics of patients and the outcome variables are shown in Table 1.

**Table 1:** Baseline characteristics of the whole population (*n *= 362) at admission to the ICU

General characteristic	Value
Demographics	
Age (years)	63.4 ± 16.5
Sex (male)	232 (64%)
Current smoking	110 (31%)
Current alcohol abuse	77 (22%)
Previous antibiotic	67 (21%)
Influenza vaccine	140 (47%)
Pneumococcal vaccine	43 (14%)
Inhaled corticosteroid	89 (25%)
Systemic corticosteroid	23 (7%)
Co-morbidity	
Chronic respiratory disease	134 (37%)
Chronic obstructive pulmonary disease	59 (16%)
Asthma	21 (6%)
Bronchiectasis	9 (3%)
Other	45 (12%)
Chronic cardiovascular disease	50 (14%)
Diabetes mellitus	70 (20%)
Neurological disease	68 (19%)
Chronic renal disease	23 (6%)
Chronic liver disease	23 (6%)
Clinical findings	
Fever	281 (78%)
Diastolic blood pressure (mmHg)	70 (21)
Systolic blood pressure (mmHg)	120 (50)
Laboratory findings	
Creatinine (mg/dl)	1.1 (0.7)
C-reactive protein level (mg/dl)	22.4 (20.8)
White blood cell count (10^9 ^cells/l)	13.4 (10.5)
Platelet count (10^9 ^platelets/l)	235.0 (127.0)
Oxygen saturation (%)	91.4 (8.5)
PaO_2_/FIO_2_	247.6 (99.5)
Pneumonia severity index	
I to III	96 (27%)
IV	129 (37%)
V	126 (36%)
Bacteraemia	63 (18%)
Multilobar infiltration	159 (44%)
Pleural effusion	79 (22%)
Severe community-acquired pneumonia	201 (66%)
Mechanical ventilation	135 (44%)
Septic shock	72 (20%)
ARDS criteria	26 (7%)
Length of hospital stay (days)	11.0 (9.0)
Thirty-day mortality	37 (10%)

Data presented as mean ± standard deviation, *n *(%) or median (interquartile range). ARDS, acute respiratory distress syndrome; PaO_2_/FIO_2_, arterial oxygen tension to inspired oxygen fraction ratio. Other respiratory diseases include sequelae of pulmonary tuberculosis, pulmonary hypertension and interstitial lung disease.

Among the 67 patients who had received antimicrobial treatment prior to hospital admission, the median duration of treatment was 2.8 days. The types of antibiotics received were: 25 (8%) β-lactams, 20 (6%) fluoroquinolones, six (2%) macrolides and 16 (5%) unknown.

### Aetiology

The specimens obtained included blood cultures from 330 (91%) patients, urine from 345 (95%) patients, acute and follow-up sera from 150 (41%) patients, sputum from 285 (79%) patients, bronchoscopically obtained lower respiratory secretions from 84 (23%) patients, pleural fluid in 62 (17%) patients, and nasopharyngeal and oropharyngeal swabs from 180 (50%) patients.

The aetiology of CAP could be established in 196 (54%) ICU patients. The proportion of patients with defined aetiology was higher in those with available lower respiratory tract samples, which included sputum and bronchoscopically obtained secretions (Table 2). Patients with lower respiratory tract samples were more severe, assessed by higher PSI risk classes, more frequent septic shock, ARDS criteria and the need for mechanical ventilation.

**Table 2:** Characteristics of patients with and without low respiratory tract samples

Characteristic	With samples (*n *= 86)	Without samples (*n *= 276)	*P *value
Age (years)	64.4 ± 16.7	63.0 ± 16.4	0.39
Sex (male)	63 (73.3%)	169 (61.2%)	
Age ≥ 65 years	47 (54.7)	149 (54)	0.91
Pneumonia severity index			0.049
I to III	13 (15.3%)	83 (31.2%)	
IV	26 (30.6%)	103 (38.7%)	
V	46(54.1%)	80 (30.1%)	
Bacteraemia	18 (21.4%)	45 (17.4%)	0.40
Multilobar infiltration	46 (53.5%)	113 (40.9%)	0.041
Severe community-acquired pneumonia	66 (83.5%)	135 (59.2%)	0.001
Mechanical ventilation	60(81.1%)	75 (32.1%)	0.001
ARDS criteria	17 (19.8%)	9 (33.3%)	0.001
Septic shock	30 (34.9%)	42 (15.3%)	0.001
Length of hospital stay (days)	24.7 (20.7)	12.8 (10.5)	< 0.001
Thirty-day mortality	19 (22.1%)	18 (6.5%)	0.001
Aetiology	60(70%)	136 (49%)	0.002
*Streptococcus pneumoniae*	29 (48.3%)	93 (68.4%)	
*Staphylococcus aureus*	3 (5%)	4 (2.9%)	
MRSA	8 (13.3%)	6 (4.4%)	
*Legionella pneumophila*	1 (1.7%)	10 (7.4%)	
*Chlamydophila pneumophila*	1 (1.7%)	5 (3.7%)	
*Haemophilus influenzae*	5 (8.3%)	3 (2.2%)	
Virus	10 (16.7%)	21(15.4%)	
*Coxiella burnetii*	1 (1.7%)	2 (1.5%)	
*Streptococcus viridans*	1 (1.7%)	0	
*Mycoplasma pneumoniae*	4 (6.7%)	2 (1.5%)	
*Pseudomonas aeruginosa*	7 (11.7%)	7 (5.1%)	
Gram-negative enteric bacilli	5 (8.3%)	5 (3.7%)	

Data presented as mean ± standard deviation, *n *(%) or median (interquartile range). ARDS, acute respiratory distress syndrome; MRSA, methicillin-resistant *Staphylococcus aureus*.

Monomicrobial infection was detected in 157 cases and polymicrobial infection in 39 cases (11% of the overall population and 20% of those with defined aetiology only), with two pathogens isolated in 33 cases and three pathogens in six cases. As shown in Table 3, the most frequently identified pathogens were *S. pneumoniae*, respiratory viruses, *P. aeruginosa*, methicillin-resistant *Staphylococcus aureus *(MRSA), Gram-negative enteric bacilli (GNEB) and *L. pneumophila*.

**Table 3:** Distribution of the causative microorganisms identified in 196 patients with community-acquired pneumonia

Microorganism	Monomicrobial aetiology (*n *= 157)	Polymicrobial aetiology (*n *= 39)	*P *value	Two pathogens (*n *= 33)	Three pathogens (*n *= 6)
*Streptococcus pneumoniae*	94 (60)	28 (72)	0.17	23 (70)	5 (83)
*Streptococcus pyogenes*	1 (1)	1 (3)	0.36	1 (3)	-
*Streptococcus viridans*^a^	-	1 (3)	0.20	1 (3)	-
*Staphylococcus aureus *(MSSA)	4 (3)	3 (8)	0.12	2 (6)	1 (17)
*Staphylococcus aureus *(MRSA)	5 (3)	9 (23)	< 0.001	5 (15)	4 (67)
*Haemophilus influenzae*	4 (3)	4 (10)	0.029	3 (9)	1 (17)
*Moraxella catarrhalis*	1 (1)	2 (5)	0.041	1 (3)	1 (17)
Gram-negative enteric bacilli^b^	6 (4)	7 (18)	0.002	5 (15)	2 (33)
*Pseudomonas aeruginosa*	6 (4)	8 (21)	< 0.001	6 (18)	2 (33)
Respiratory viruses	16 (10)	15 (39)	< 0.001	14 (42)	1 (17)
Rhinovirus	2 (1)	2 (5)	0.13	2 (6)	-
Adenovirus	1 (1)	1 (3)	0.36	1 (3)	-
Respiratory syncitial virus	2 (1)	2 (5)	0.13	2 (6)	-
Influenza virus A	10 (6)	9 (23)	0.002	8 (24)	1 (17)
Influenza virus B	1 (1)	1 (3)	0.36	1 (3)	-
*Legionella pneumophila*	10 (6)	1 (3)	0.36	1 (3)	-
Atypical	10 (6)	5 (13)	0.18	4 (12)	1 (17)
*Mycoplasma pneumoniae*	4 (3)	2 (5)	0.40	2 (6)	-
*Chlamydophila pneumoniae*	4 (3)	2 (5)	0.40	1 (3)	1 (17)
*Coxiella burnetii*	2 (1)	1 (3)	0.49	1 (3)	-

Data presented as *n *(%). Percentages refer to the total number of patients of each group (monomicrobial vs. polymicrobial). The most frequent combinations in cases with two pathogens were *S. pneumoniae *with respiratory viruses (11 cases), *P. aeruginosa *(three cases), and *H. influenzae*, Gram-negative enteric bacilli and atypicals in two cases each. The most frequent combination in cases with three pathogens was *S. pneumoniae*, Gram-negative enteric bacilli and methicillin-resistant *Staphylococcus aureus *(MRSA) in two cases. *P *value refers to a statistical comparison of cases with monomicrobial aetiology and polymicrobial aetiology. MSSA, methicillin-susceptible *Staphylococcus aureus*. ^a^*S. viridans *isolated from a pleural fluid specimen. ^b^Including *Escherichia coli, Klebsiella pneumoniae, Serratia marcescens*.

### Comparison of the monomicrobial and polymicrobial aetiology

Patients with polymicrobial aetiology had previously received antibiotics less frequently, had a higher proportion of chronic respiratory and neurological diseases, less frequently presented fever at admission, had higher rates of PSI risk class V, had severe CAP according to the IDSA/ATS definition, and fulfilled ARDS criteria. The length of hospital stay and hospital mortality tended to be higher in these patients (Table 4).

**Table 4:** Characteristics of patients with defined aetiology, comparing monomicrobial and polymicrobial pneumonia

Variable	Monomicrobial CAP (*n *= 157)	Polymicrobial CAP (*n *= 39)	*P *value
Demographics			
Age (years)	61.6 ± 17.7	63.5 ± 14.1	0.48
Sex (male)	106 (68%)	23 (59%)	0.31
Current smoking	46 (30%)	13 (35%)	0.53
Current alcohol abuse	32 (21%)	12 (33%)	0.11
Previous antibiotic	30 (22%)	2 (7%)	0.051
Influenza vaccine	50 (39%)	9 (35%)	0.65
Pneumococcal vaccine	20 (16%)	2 (8%)	0.29
Inhaled corticosteroid	29 (19%)	11 (31%)	0.10
Systemic corticosteroid	11 (7%)	4 (12%)	0.34
Co-morbidity			
Chronic respiratory disease	50 (32%)	21 (54%)	0.011
Chronic cardiovascular disease	21 (14%)	1 (3%)	0.068
Diabetes mellitus	26 (17%)	4 (11%)	0.40
Neurological disease	19 (12%)	9 (25%)	0.052
Chronic renal disease	10 (7%)	2 (6%)	0.84
Chronic liver disease	9 (6%)	5 (13%)	0.11
Clinical findings			
Fever	132 (84%)	27 (69%)	0.034
Diastolic blood pressure (mmHg)	68.0 (20.0)	70.0 (22.0)	0.38
Systolic blood pressure (mmHg)	120.0 (48.0)	121.0 (63.0)	0.23
Laboratory findings			
Creatinine (mg/dl)	1.2 (0.8)	1.3 (0.7)	0.57
C-reactive protein level (mg/dl)	25.2 (20.3)	26.5 (11.6)	0.24
White blood cell count (10^9 ^cells/l)	13.1 (12.1)	9.5 (13.4)	0.019
Oxygen saturation (%)	93 (7.9)	92.0 (7.3)	0.78
PaO_2_/FIO_2_	254.3 (84.6)	247.6 (104.8)	0.38
Inappropriate empirical treatment	15 (10%)	15 (39%)	< 0.001
Pneumonia severity index			
I to III	44 (29%)	10 (28%)	0.89
IV	60 (40%)	8 (22%)	0.053
V	48 (32%)	18 (50%)	0.037
Severe CAP	94 (68%)	31 (86%)	0.029
Bacteraemia	47 (32%)	14 (39%)	0.45
Multilobar infiltration	74 (47%)	27 (69%)	0.013
Pleural effusion	36 (23%)	9 (24%)	0.94
Mechanical ventilation	60 (43%)	21 (62%)	0.047
ARDS criteria	6 (4%)	12 (31%)	< 0.001
Septic shock	38 (24%)	14 (36%)	0.14
Length of hospital stay (days)	12.0 (11.0)	15.0 (10.0)	0.082
Thirty-day mortality	17 (11%)	8 (21%)	0.10

Data presented as mean ± standard deviation, *n *(%) or median (interquartile range). ARDS, acute respiratory distress syndrome; CAP, community-acquired pneumonia; PaO_2_/FIO_2_, arterial oxygen tension to inspired oxygen fraction ratio. Other respiratory diseases include sequelae of pulmonary tuberculosis, pulmonary hypertension and interstitial lung disease.

As regards the aetiologic pathogens, the proportion of respiratory viruses-particularly influenza A, MRSA, *P. aeruginosa*, GNEB, *Haemophilus influenzae *and *Moraxella catarrhalis *- were more frequently isolated in patients with polymicrobial pneumonia, without differences in the remaining pathogens (Table 3).

### Empirical antibiotic therapy

Data on antibiotic treatment were available in 347 (96%) patients. The most frequent regimens were fluoroquinolones plus β-lactam (*n *= 217, 63%), β-lactam plus macrolide (*n *= 73, 21%), fluoroquinolone monotherapy (*n *= 39, 11%) and β-lactam monotherapy (*n *= 18, 5%). These regimens were similarly administered in patients with monomicrobial or polymicrobial aetiology.

The empirical antibiotic treatment was more frequently inappropriate in patients from the polymicrobial aetiology group (Table 4). When respiratory viruses were not taken into account, the pathogens most frequently associated with inadequate treatment were MRSA in 10 cases, and *S. pneumoniae, P. aeruginosa *and GNEB in nine cases each. None of our patients received antiviral therapy.

### Predictors of polymicrobial aetiology

Several variables were significantly associated with polymicrobial pneumonia in the univariate logistic regression analyses (Table 5). Among these variables, chronic respiratory disease and ARDS criteria at hospital admission were independent predictors of polymicrobial aetiology in the multivariate analysis.

**Table 5:** Univariate and multivariate logistic regression analyses of predictors of polymicrobial pneumonia

Variable	Univariate analysis			Multivariate analysis^a^		
	Odds ratio	95% CI	*P *value	Adjusted odds ratio	95% CI	*P *value
Previous antibiotic	0.25	0.06 to 1.11	0.068	**-**	**-**	**-**
Neurological disease	2.39	0.98 to 5.83	0.057			
Chronic respiratory disease	2.50	1.22 to 5.10	0.012	2.86	1.31 to 6.25	0.008
Fever	0.43	0.19 to 0.95	0.037	-	-	-
WBC (+10 × 10^9 ^cells/l)^b^	0.61	0.38 to 0.98	0.041	**-**	***-***	**-**
Multilobar infiltration	2.52	1.19 to 5.34	0.015	-	-	-
Mechanical ventilation	2.15	1.00 to 4.64	0.050	-	-	**-**
ARDS criteria	11.11	3.84 to 32.14	< 0.001	12.31	4.08 to 37.12	< 0.001

ARDS, acute respiratory distress syndrome; CI, confidence interval; WBC, white blood cells. ^a^Hosmer-Lemeshow goodness-of-fit test, *P *= 0.55. ^b^Increase by 10 × 10^9 ^cells/l.

### Predictors of hospital mortality

The univariate logistic regression analyses revealed several variables significantly associated with hospital mortality (Table 6). Although polymicrobial pneumonia (that is, two or more pathogens identified) was associated with increased mortality compared with the absence of defined aetiology, the differences between monomicrobial and polymicrobial aetiology were not significant, as shown in Table 4.

**Table 6:** Univariate and multivariate logistic regression analysis of predictors of mortality

Variable	Univariate analysis			Multivariate analysis^a^		
	Odds ratio	95% CI	*P *value	Adjusted odds ratio	95% CI	*P *value
Age ≥ 65 years	2.49	1.17 to 5.32	0.018	3.06	1.27 to 7.41	0.013
Diabetes mellitus	2.25	1.06 to 4.76	0.034	-	-	-
Neurological disease	2.04	0.95 to 4.38	0.068	2.63	1.07 to 6.48	0.036
Chronic liver disease	5.63	2.20 to 14.39	< 0.001	8.99	2.91 to 27.77	< 0.001
Fever	0.49	0.24 to 1.01	0.053	-	-	-
Pneumonia severity index IV to V	4.77	1.43 to 15.91	0.011	-	-	-
Multilobar infiltration	2.28	1.13 to 4.60	0.021	-	-	-
Number of pathogens identified^b^			0.055	-	-	-
None	1	-	-			
Monomicrobial	1.56	0.72 to 3.38	0.26	-	-	-
Polymicrobial	3.31	1.25 to 8.77	0.016	-	-	-
Inappropriate empiric treatment	11.23	4.44 to 28.38	< 0.001	10.79	3.97 to 29.30	< 0.001

^a^Hosmer-Lemeshow goodness-of-fit test, *P *= 0.81. ^b^The *P *value corresponds to differences between the three groups (none, one or more than one pathogen). The odds ratio and 95% confidence interval (CI) of monomicrobial and polymicrobial pneumonia are related to cases with no pathogen identified.

Among these variables, age ≥ 65 years, neurological disease, chronic liver disease and inappropriate antimicrobial treatment were independently associated with increased hospital mortality in the multivariate analysis.

## Discussion

Polymicrobial aetiology was found in 11% of all patients with CAP admitted to the ICU, 20% considering those with defined aetiology only. Although *S. pneumoniae *was the most frequent pathogen in both groups, we found MRSA, *P. aeruginosa*, GNEB, *H. influenzae, M. catarrhalis *and respiratory viruses more frequently identified in polymicrobial pneumonia than in monomicrobial pneumonia. Chronic respiratory disease and ARDS criteria were independent predictors of polymicrobial aetiology. Although an independent predictor of hospital mortality such as inappropriate treatment was more frequent in the polymicrobial aetiology group, the trend for higher hospital mortality in patients from this group was not statistically significant.

In general populations of hospitalised patients with CAP, we have previously reported lower rates of polymicrobial pneumonia (5%) [3,12] than in this series of ICU patients. Other studies on patients with CAP found 5.7% and 38.4% rates of polymicrobial aetiology in their series [4,5]. These wide variations might be explained by differences in the populations studied, epidemiological settings, rate of antimicrobial pretreatment, microbiological workup and definitions of aetiology. A typical limitation of many studies dealing with microbial aetiology in CAP is that not all microbiological tests are applied systematically for all patients. This issue means that the real frequency of polymicrobial aetiologies could possibly be higher if a complete microbiological investigation was performed in all cases. In view of these methodological problems, it seems difficult to indicate precisely the extent of the problem of polymicrobial aetiology. Analysing the potential impact of polymicrobial aetiology is therefore more important, particularly in the most severely ill patients and in those at highest risk of death.

*S. pneumoniae *was not only the most frequent pathogen but also by far the most frequent co-pathogen in polymicrobial infections. This finding underlines the importance of pneumococcal coverage in any initial antimicrobial treatment regimen. The most frequent polymicrobial pattern was *S. pneumoniae *and viral infection, particularly influenza virus. Pneumococci have been identified as the most frequent bacterial superinfection in both seasonal [13] and novel H1N1 [14,15] influenza virus-associated pneumonia.

Interestingly, whereas *S. pneumoniae *was by far the most frequent single pathogen, the rate of this pathogen was similar among patients with monomicrobial aetiology and those with polymicrobial aetiology. Among the pathogens more frequently identified in polymicrobial pneumonia, respiratory viruses were the most frequent. We did not find that polymicrobial aetiology was associated with higher mortality. Viruses were the most frequent microorganisms associated with polymicrobial aetiology. Except for influenza A H1N1, viruses are not a cause of excess mortality-as recently pointed out by two recent studies [13,16]. The role of viruses in the aetiology of pneumonia is unclear, since they may be regarded either as primary infection or, with bacteria, as representing superinfection [17]. None of our patients received antiviral treatment. We feel that at least during the influenza season, however, patients could benefit from antiviral treatment.

The role of MRSA in CAP is limited in Europe, even if patients meeting criteria for healthcare-associated pneumonia remain included [18]. Although for our series we excluded patients with healthcare-associated pneumonia, the frequent association of this pathogen with severe underlying illness [19] may explain the higher rate of this pathogen in the polymicrobial aetiology group, since these patients were more severe at admission than those with monomicrobial aetiology. The exact role of MRSA in polymicrobial CAP is difficult to assess, however, because even a high bacterial load of MRSA may still represent colonisation rather than infection [20]. The higher rate of *P. aeruginosa *and GNEB in polymicrobial pneumonia may also be related to the higher rate of chronic respiratory diseases in this group, since identification of these pathogens occurs more frequently in those with chronic lung disease [21]. As for MRSA, the identification of *P. aeruginosa *does not necessarily mean this is the causative pathogen of acute exacerbation in all chronic obstructive pulmonary disease patients colonised by the pathogen [22], and similarly MRSA eventually may represent colonisation rather than infection in patients with pneumonia.

We identified chronic respiratory disease and ARDS criteria as independent predictors of polymicrobial aetiology. In chronic obstructive pulmonary disease, this finding can be explained by the previous colonisation of different bacteria these patients may have in their lower airways. On the contrary, ARDS may be the consequence of a mixed infection with higher pulmonary insult. In both chronic obstructive pulmonary disease and ARDS with severe CAP, our recommendation is to give a broad empirical antibiotic treatment from the beginning of therapy because mixed infections are more frequent [23,24].

A relevant issue in polymicrobial aetiology of severe CAP refers to its potential prognostic implications. We found a strong association between polymicrobial aetiology and initial inappropriate antimicrobial treatment, which in turn was an independent predictor of increased hospital mortality. Inappropriate empiric treatment has already been associated with poor outcome in patients with severe infections [25,26]. Although crude mortality was near double in patients with polymicrobial aetiology, this difference did not reach statistical significance-probably due to the insufficient number of patients included. These results indicate that the impact of initial inappropriate antimicrobial treatment is crucial for survival, and that polymicrobial aetiology is an important determinant for such inappropriateness.

To the best of our knowledge, this is the first study addressing the issue of multiple aetiologies of CAP in a large population of ICU patients. We decided to include all patients admitted to the ICU regardless of whether they met IDSA/ATS severity criteria. We think that clinical decisions for ICU admission may be valid, while the IDSA/ATS severity criteria have proven to be overly sensitive [1,12].

Several limitations have to be addressed. First, the complete diagnostic workup and microbiological sampling could not be applied in every patient. Second, the true incidence of polymicrobial aetiology may be underestimated since 21% patients had received prior antimicrobial treatment. Finally, viral infections may have been missed since paired serology is frequently not available in nonsurvivors. We did not include molecular techniques such as PCR for bacterial detection. We believe that the systematic use of qualitative and quantitative PCR for the diagnosis of respiratory infections may increase substantially the number of identified bacterial pathogens [7,27]. Moreover, these new techniques could play a crucial role in the determination of the clinical impact of polymicrobial aetiology in CAP. Unfortunately, the use of molecular techniques is not yet part of the routine diagnostic workup in CAP.

## Conclusions

Polymicrobial aetiology is a frequent finding in patients with CAP admitted to the ICU. Our data support the potential implication of polymicrobial pneumonia in the outcome of patients related to an increased risk of inappropriate antimicrobial treatment, and suggests the importance of an extensive microbiological testing in very severe CAP patients since the CAP may be caused by more than one aetiology.

The most important clinical implication of the identified predictors of polymicrobial aetiology is to emphasise the use of broad-spectrum antimicrobial treatment in these groups of patients.

## Key messages

• Polymicrobial aetiology is frequent among patients with CAP admitted to the ICU and may result in inappropriate empiric antimicrobial treatment.

• Polymicrobial aetiology of CAP should be suspected in the presence of chronic respiratory disease or criteria for ARDS.

• If antimicrobial treatment is appropriate, polymicrobial aetiology does not result in increased hospital mortality from severe CAP.

## Abbreviations

ARDS: acute respiratory distress syndrome; BAL: bronchoalveolar lavage; CAP: community-acquired pneumonia; GNEB: Gram-negative enteric bacilli; IDSA/ATS: Infectious Disease Society of America/American Thoracic Society; MRSA: methicillin-resistant *Staphylococcus aureus*; PCR: polymerase chain reaction; PSI: pneumonia severity index; RT: reverse transcriptase.

## Competing interests

The authors declare that they have no competing interests.

## Authors' contributions

CC is the main author of the paper; she reviewed the study data and realised the statistical analysis, edited the main body of the manuscript, and contributed to supervising the collection of clinical, radiological and microbiological data. SE contributed to conception of the project and database design. MF contributed to results analysis and interpretation, and to editing the final manuscript. EP contributed to data analysis and drafting the original manuscript, and to supervising the collection of clinical, radiological and microbiological data. AG realised the statistical analysis of the study. JPdlB supervised the microbiological studies. JM supervised the collection of epidemiologic and microbiological data. AT led the study group, contributed to conception of the project design and contributed to the final study, being the guarantor of the entire manuscript. All authors read and approved the manuscript for publication.

## References

[B1] Ewig S Woodhead M Torres A Towards a sensible comprehension of severe community-acquired pneumonia Intensive Care Med 2011 37 214 223 10.1007/s00134-010-2077-0 21080155

[B2] Gutierrez F Masia M Rodriguez JC Mirete C Soldan B Padilla S Hernandez I Royo G Martin-Hidalgo A Community-acquired pneumonia of mixed etiology: prevalence, clinical characteristics, and outcome Eur J Clin Microbiol Infect Dis 2005 24 377 383 10.1007/s10096-005-1346-2 15931452

[B3] de Roux A Ewig S Garcia E Marcos MA Mensa J Lode H Torres A Mixed community-acquired pneumonia in hospitalised patients Eur Respir J 2006 27 795 800 10.1183/09031936.06.00058605 16585087

[B4] Lieberman D Schlaeffer F Boldur I Horowitz S Friedma MG Leiononen M Horovitz O Manor E Porath A Multiple pathogens in adult patients admitted with community-acquired pneumonia: a one year prospective study of 346 consecutive patients Thorax 1996 51 179 184 10.1136/thx.51.2.179 8711652 PMC473032

[B5] Lim WS Macfarlane JT Boswell TC Harrison TG Rose D Leinonen M Saikku P Study of community acquired pneumonia aetiology (SCAPA) in adults admitted to hospital: implications for management guidelines Thorax 2001 56 296 301 10.1136/thorax.56.4.296 11254821 PMC1746017

[B6] Cilloniz C Ewig S Polverino E Marcos MA Esquinas C Gabarrus A Mensa J Torres A Microbial aetiology of community-acquired pneumonia and its relation to severity Thorax 2011 66 340 346 10.1136/thx.2010.143982 21257985

[B7] Menendez R Cordoba J de la Cuadra P Cremades MJ Lopez-Hontagas JL Salavert M Gobernado M Value of the polymerase chain reaction assay in noninvasive respiratory samples for diagnosis of community-acquired pneumonia Am J Respir Crit Care Med 1999 159 1868 1873 10351932 10.1164/ajrccm.159.6.9807070

[B8] Fine MJ Auble TE Yealy DM Hanusa BH Weissfeld LA Singer DE Coley CM Marrie TJ Kapoor WN A prediction rule to identify low-risk patients with community-acquired pneumonia N Engl J Med 1997 336 243 250 10.1056/NEJM199701233360402 8995086

[B9] Mandell LA Wunderink RG Anzueto A Bartlett JG Campbell GD Dean NC Dowell SF File TM Musher DM Niederman MS Torres A Whitney CG Infectious Diseases Society of America/American Thoracic Society consensus guidelines on the management of community-acquired pneumonia in adults Clin Infect Dis 2007 44 Suppl 2 S27 S72 17278083 10.1086/511159 PMC7107997

[B10] European Committee on Antimicrobial Susceptibility Testing http://www.eucast.org/clinical_breakpoints/

[B11] Hosmer D Lemeshow S Applied Logistic Regression 1989 New York: Wiley

[B12] Liapikou A Ferrer M Polverino E Balasso V Esperatti M Piner R Mensa J Luque N Ewig S Menendez R Niederman MS Torres A Severe community-acquired pneumonia: validation of the Infectious Diseases Society of America/American Thoracic Society guidelines to predict an intensive care unit admission Clin Infect Dis 2009 48 377 385 10.1086/596307 19140759

[B13] von Baum H Schweiger B Welte T Marre R Suttorp N Pletz MW Ewig S How deadly is seasonal influenza associated pneumonia? The German Competence Network for Community-acquired pneumonia (CAPNETZ) Eur Respir J 2010 37 1151 1157 20817703 10.1183/09031936.00037410

[B14] Centres for Disease Control and Prevention Bacterial coinfections in lung tissue specimens from fatal cases of 2009 pandemic influenza A (H1N1)-United States, May-August 2009 MMWR Morb Mortal Wkly Rep 2009 58 1071 1074 19798021

[B15] Martin-Loeches I Sanchez-Corral A Diaz E Granada RM Zaragoza R Villavicencio C Albaya A Cerda E Catalan RM Luque P Paredes A Navarrete I Rello J Rodriguez A Community-acquired respiratory coinfection in critically ill patients with pandemic 2009 influenza A(H1N1) virus Chest 2011 139 555 562 10.1378/chest.10-1396 20930007

[B16] Riquelme R Torres A Rioseco ML Ewig S Cilloniz C Riquelme M Inzunza C Polverino E Gomez Y Marcos MA Contreras C Gabarrus A Fasce R Influenza pneumonia: a comparison between seasonal influenza virus and the H1N1 pandemic Eur Respir J 2011 38 106 111 10.1183/09031936.00125910 21109555

[B17] McCullers JA Insights into the interaction between influenza virus and pneumococcus Clin Microbiol Rev 2006 19 571 582 10.1128/CMR.00058-05 16847087 PMC1539103

[B18] Carratala J Mykietiuk A Fernandez-Sabe N Suarez C Dorca J Verdaguer R Manresa F Gudiol F Health care-associated pneumonia requiring hospital admission: epidemiology, antibiotic therapy, and clinical outcomes Arch Intern Med 2007 167 1393 1399 10.1001/archinte.167.13.1393 17620533

[B19] Lim WS Baudouin SV George RC Hill AT Jamieson C Le J Macfarlane JT Read RC Roberts HJ Levy ML Wani M Woodhead MA BTS guidelines for the management of community acquired pneumonia in adults: update 2009 Thorax 2009 64 Suppl 3 iii1 iii55 10.1136/thx.2009.121434 19783532

[B20] Dallas J Skrupky L Abebe N Boyle WA Kollef MH Ventilator-associated tracheobronchitis in a mixed surgical and medical ICU population Chest 2011 139 513 518 10.1378/chest.10-1336 20724738

[B21] Torres A Dorca J Zalacaín R Bello S El-Ebiary M Molinos L Arevalo M Blanquer J Celis R Iriberri M Prats E Fernandez R Irigaray R Serra J Community-acquired pneumonia in chronic obstructive pulmonary disease. Spanish Multicenter Study Am J Respir Crit Care Med 1996 154 1456 1461 8912764 10.1164/ajrccm.154.5.8912764

[B22] Murphy TF *Pseudomonas aeruginosa *in adults with chronic obstructive pulmonary disease Curr Opin Pulm Med 2009 15 138 142 10.1097/MCP.0b013e328321861a 19532029

[B23] Sethi S Maloney J Grove L Wrona C Berenson CS Airway inflammation and bronchial bacterial colonization in chronic obstructive pulmonary disease Am J Respir Crit Care Med 2006 173 991 998 10.1164/rccm.200509-1525OC 16474030 PMC2662918

[B24] Bauer TT Ewig S Rodloff AC Muller EE Acute respiratory distress syndrome and pneumonia: a comprehensive review of clinical data Clin Infect Dis 2006 43 748 756 10.1086/506430 16912951 PMC7107989

[B25] Kumar A Ellis P Arabi Y Roberts D Light B Parrillo JE Dodek P Wood G Kumar A Simon D Peters C Ahsan M Chateau D Initiation of inappropriate antimicrobial therapy results in a fivefold reduction of survival in human septic shock Chest 2009 136 1237 1248 10.1378/chest.09-0087 19696123

[B26] Niederman MS Appropriate use of antimicrobial agents: challenges and strategies for improvement Crit Care Med 2003 31 608 616 10.1097/01.CCM.0000050464.70382.D6 12576973

[B27] Palacios G Hornig M Cisterna D Savji N Bussetti AV Kapoor V Hui J Tokarz R Briese T Baumeister E Lipkin WI *Streptococcus pneumoniae *coinfection is correlated with the severity of H1N1 pandemic influenza PLoS One 2009 4 e8540 10.1371/journal.pone.0008540 20046873 PMC2795195

